# Regucalcin expression profiles in veal calf testis: validation of histological and molecular tests to detect sex steroids illicit administration

**DOI:** 10.7717/peerj.10894

**Published:** 2021-02-19

**Authors:** Alessandro Benedetto, Elena Biasibetti, Chiara Beltramo, Valentina Audino, Simone Peletto, Elena Maria Bozzetta, Marzia Pezzolato

**Affiliations:** Istituto Zooprofilattico Sperimentale del Piemonte, Liguria e Valle d’Aosta, Turin, Italy

**Keywords:** Regucalcin, Growth promoters, Immunohistochemistry, Real Time PCR, FFPE tissues

## Abstract

**Background:**

Sex steroids administration in meat producing animals is forbidden within the EU to preserve consumers’ safety, but continuous monitoring to identify resurgence of their misuse is needed. Among biomarkers related to sex steroids abuse in veal calves the regucalcin (RGN) mRNA perturbations in testis have been described in RNA*later* samples. To setup novel diagnostic method, to update current tests available in National Residue Control Plans (NRCPs) and in legal dispute when illicit practices on farm animals are suspected, the reliability of RGN profiling was assessed by histological and molecular techniques.

**Methods:**

Formalin fixed paraffin embedded (FFPE) testis samples, chosen being the most effective preservation strategy adopted by histological NRCPs and allowing easier retrospective analysis if required by legal disputes, were analyzed from veal calves treated with nandrolone, 17β-estradiol and a cocktail of the two hormones. RGN levels were determined by quantitative Real Time PCR and Immunohistochemistry assays. Test performances were assessed and compared by multiple ROC curves.

**Results:**

Both tests resulted sensitive and specific, allowing to enrich, in future field investigation, novel integrated diagnostic protocols needed to unveil sex steroid abuse.

**Discussion:**

Developed RT-qPCR and IHC methods confirmed RGN as a useful and robust biomarker to detect illegal administration of sex steroid hormones in veal calves. The developed methods, successfully applied to ten years old FFPE blocks, could allow both retrospective analysis, when supplementary investigations are requested by authorities, and future implementation of current NRCPs.

## Introduction

The use of sex steroids in food producing animals is forbidden within the EU to preserve consumers’ safety. Notwithstanding, use of prohibited designer drugs which are otherwise unmonitored by National Residue Control Plans (NRCP) was highlighted in the annual technical report of the European Food Safety Authority (EFSA) on drug residue analysis (EFSA, 2020).

Moreover, cocktails of drugs can also be used to escape official controls: the combined use at low concentration of different molecules gains sum effect and determines low residues levels, often hardly identifiable by routine monitoring also when performed by analytical methods like High Performance Liquid Chromatography tandem Mass Spectrometry (HPLC-MS).

Illicit growth promoters’ cocktails are known to cause, together with increased meat production, several perturbations in different tissue biomarkers that can be exploited to setup diagnostic screening tools improving the efficacy of surveillance systems (Guglielmetti et al., 2014; Pezzolato et al., 2016). Recently it has been shown that sex steroids induce a reduction of regucalcin (RGN) expression in calves testicles (Starvaggi Cucuzza et al., 2014). Such an effect can be detected by monitoring the expression of RGN at different levels (mRNA, proteins), thus different approaches have been proposed (Benedetto et al., 2019; Starvaggi Cucuzza et al., 2017).

However, their applicability as suitable diagnostic tools for daily testing of samples collected from slaughterhouses in the frame of NRCPs (EFSA, 2013) could be difficult for intrinsic characteristic of slaughtering procedures. Indeed, several different sampling types are needed for official meat inspection activities, requiring fixed execution times, specific samples pre-treatment and consequent storage conditions, slowing down and overloading slaughter chain, therefore, creating added cost and sometimes to the point of discouraging their proper execution. A simplification of sampling and sample management could therefore facilitate the application of molecular biomarkers analysis in daily routine, as already shown in such context by previous studies (Benedetto et al., 2020).

Considering recent implementations introduced for nucleic acid analysis on formalin fixed paraffin embedded (FFPE) tissues (Zeka et al., 2016) the possibility to perform molecular retrospective analysis on FFPE testis seems feasible.

Furthermore, when positive results for suspected use of steroids and/or steroids-like activity compounds are obtained by the untargeted histological screening approach applied in Italy according to the Residue Control Plans in place, further confirmatory tests are required.

The aim of this work was therefore to compare RGN mRNA with RGN protein levels on paraffin fixed testis tissue, in order to setup and combine these diagnostic tests for sex steroid hormones detection in veal calves in the frame of NRCPs. The new diagnostic protocol set up could also allow retrospective analyses on archival material when needed, without the complications and the extra costs expected for conventional RNA preservation procedures on collected samples (trained staff at abattoir plants, RNAse free devices and containers, RNA*later* reagents, preliminary storage at 4 °C for 24 h, removal of excess reagents before storage at −20 °C, etc.).

## Material and Methods

### Samples

Archived paraffin blocks of bovine testis samples coming from a previous animal trial were retrieved from tissue bank of National Reference Center for Biological screening of anabolic substances in producing animals (Centro per le Indagini Biologiche sugli Anabolizzanti animali—CIBA) and analysed. Specifically, 40 testis blocks from calves similar in age (7 months old on average) treated with one of the four different protocols applied (see below) were tested.

All animals were treated from the sixth to the seventh month of age and slaughtered after a withdrawal period of at least 20 days. Treatment schedules and groups sizes were defined as follow: nandrolone (*n* = 10; 50 mg/head/week; four intramuscular injections), 17β-estradiol (*n* = 10; 5 mg/head/week; four intramuscular injections), a cocktail of both nandrolone and 17 β-estradiol (*n* = 10; 50 + 5 mg/head/week, four intramuscular injections) and 10 untreated control animalsThe experiment was authorised by the Italian Ministry of Health and the Ethics Committee of the University of Turin and was carried out according to European Economic Community Council Directive 86/ 609, recognised and adopted by the Italian Government (DLgs 27∕01∕1992 no. 116).

### Primer design

To overcome the issue of low RNA integrity due to formalin cross links in FFPE samples, primers and probes for RGN and Peptidylprolyl Isomerase A (PPIA) transcripts were designed to amplify the as short as possible amplicons and they were specifically targeted to the same genomic regions selected by Starvaggi Cucuzza et al. (2017) for RGN and Benedetto et al. (2020) for PPIA, in order to enhance assay sensitivity and to maintain acceptable assay specificity.

Real Time PCR Taqman MGB assays were designed with Primer Express 3.0 Software (Applied Biosystems). To further enhance test sensitivity a one-step retro-transcription and quantitative Real Time PCR (RT-qPCR) strategy was chosen: this approach based on gene specific reverse transcription and multiplex Real Time PCR has been shown by previous studies as the one of the most fast and accurate strategies for gene expression analysis on FFPE material (Zeka et al., 2016).

Preliminary checks for primers, probes and secondary structures were performed by Primer Express 3.0 software and Mfold analysis (Zuker, 2003).

### RNA isolation and quantification

Total RNA extraction from collected FFPE testis was performed using the RNeasy FFPE kit (Qiagen, Hilden, Germany) following the manufacturer’s recommendations.

Quantity of extracted RNA and residual DNA was assessed respectively by Qubit BR-RNA and HS-DNA fluorometric assay (Invitrogen, Waltham, USA) while RNA quality was checked by Bionalazyer 2100 using RNA 6000 Nano Chip (Agilent, Santa Clara, USA). RNA samples selection for downstream RT-qPCR was made considering a RIN (RNA Integrity Number) cut of 1.4, characterized, according to previous studies (Benedetto et al., 2020; Ribeiro-Silva, Zhang & Jeffrey, 2007), by a predominant RNA fragments sizeof 100 bp (67-151bp), exceeding therefore the sizes of PPIA and RGN transcript regions selected for RT-qPCR amplification.

### Multiplex One step RT-qPCR

The RGN and PPIA cDNA synthesis and PCR amplification was performed multiplexing two Taqman assays in single 20 µL reactions, containing 11 µl of QuantiNova Probe RT-PCR Master Mix (Qiagen), 0.2 µL of 100x QuantiNova RT Mix (Qiagen), a final concentration of 0.8 µM for each primer, 0.2 µM of two TaqMan probes respectively, and 400 ng of total RNA. Primers and probes of the two assays are reported in Table 1. All runs were performed on a CFX-96 Touch thermal cycler (Bio-Rad, Berkeley, USA) according to the following cycling conditions: retro-trascription step by 45 °C incubation for 10 min, followed by Taq activation at 95 °C for 5 min, 40 cycles with a denaturation step at 95 °C for 5 s and an annealing/extension step at 60 °C for 1 min. Each sample was analysed in triplicate. Standard curves used for quantification of RGN and PPIA targets at copy number level were performed with amplification of serial dilutions of reference plasmid containing the sequences of both targets. The plasmid material was produced and checked as described in a recent work of our team (Benedetto et al., 2020) and analysed with the same PCR mixes/thermal profile defined for RNA samples, replacing with an equivalent volume of water the QuantiNova reagents for Retro-transcription and genomic DNA (gDNA) depletion not required for plasmid amplification.

The same modified PCR mixes adopted for standard curves amplification were also used to test RNA samples without retro-transcription step. These negative controls are needed to check unexpected amplifications signals from RGN/PPIA amplicons cross-contamination and/or presence of targeted pseudogenes in residual DNA.

### Immunohistochemistry

Paraffin sections (3 +/-2 µm) from all the 40 veal calves’ testes samples were deparaffinised and rehydrated in graded alcohols. Heat-induced (97 °C) citrate buffer (pH 6) antigen retrieval was applied in a water bath for 30 min. Slides were then incubated with 3% hydrogen peroxide for 30 min at room temperature to inhibit endogenous peroxidase activity. Sections were incubated with anti-RGN rabbit polyclonal antibody (Sigma, Milwaukee, USA) diluted 1:100 for 60 min at room temperature. The EnVision System Kit (Dako, Glostrup, Denmark) was used for identification purposes. Diaminobenzidine-hydrogen peroxide solution (Dako) was used as chromogen and applied for 4 min. Sections were slightly counterstained with hematoxylin, dehydrated, cleared and mounted. The specificity of the staining was assessed by the omission of the primary antibody.

Digital images were obtained with a Nikon DS-Fi1 colour digital camera (Nikon Instruments, Tokyo, Japan).

A quantitative analysis of immunohistochemistry (IHC) was performed. Five digital images (5 randomly selected fields, 200x) were obtained with a Nikon DS-Fi1 colour digital camera (Nikon Instruments) for each slide and then a quantitative analysis of immunohistochemistry (IHC) was performed by NIS-Elements 4.5.

Each image was analysed with the pixel classificatory, a tool that enables the user to group together and classify a range of similar pixels in an image. After the definition of the groups of pixels to be included in phase one on the bases of colour and dimension, the algorithm was saved. Afterward all images were automatically analysed and data on percentage of phase 1 expressed as percentages of positive tissue area versus total tissue area were exported on a data sheet for statistical analysis.

### Data analysis and statistics

RGN and PPIA Cq data were analyzed using GenEx software (Multi ID) for both relative quantification (RQ) by ΔΔCq (Livak & Schmittgen, 2001) and standard curve quantification method. Normal distribution of collected data among different treatment groups was verified by Shapiro–Wilk Normality test; then multiple comparisons were performed by ANOVA (post hoc correction by the Dunnet test considered significant with *p*-value <0.05).Outlier detection for repeated measures was performed by the Grubbs’ test. For the analysis of FFPE samples by a quantitative approach alternative to ΔΔCq, the copy-ratio of RGN-PPIA was then calculated from quantified copy number of each target using a standard curve method. Main standard curves parameters such as linearity, slope, R^2^, Limit of Detection (LOD) and Limit of Quantification (LOQ) were determined as previously reported (Benedetto et al., 2020), by both full range standard curves (performed in different days on four separate runs, with five technical replicates for each tested dilution respectively), and daily curves loaded in each 96 well plate together with analyzed cDNA samples (see Supplemental Information 2). The LOQ value was intended as the LOD copy number value with a Coefficient of Variation (CV%) ≤ 25%. Finally, a receiver operator curve (ROC) was assessed to define a cut-off value with the best balance between sensitivity and specificity of the analysis for both PCR and IHC data.

Regarding IHC determinations Shapiro–Wilk test was used to test for the normality of data. The one-way Kruskal–Wallis test and Dunn post hoc test were then performed to investigate differences among the groups when the data did not follow the normal distribution. Data were expressed as median and standard deviation. Significance was declared at *p* <0.05.

## Results

Total RNA was extracted from all the 40 FFPE samples; RNA yields by fluorometric quantification ranged between 226 ng/µl and 2.41 µg/µl, while RIN ranged from 1.9 to 2.4.

**Table 1:** Oligos used for the RGN and PPIA assays. Amplicon sizes were reduced (from the original sizes of 115 bp for RGN and 95 bp for PPIA) to increase assays sensitivity on FFPE specimens.

Oligo name	Sequence	NCBI accession	Amplicon size
RGN-Forward	5′- TGGATCCCGCTGGGAGATA -3′	NM_173957	72 bp
RGN-Reverse	5′- GGCGGCGCTCCAAAA -3′		
RGN-Probe	5′- TTTGCTGGTACCATGGC -3′		
PPIA-Forward	5′-GGTTCCCAGTTTTTCATTTGCA-3′	NM_178320	69 bp
PPIA-Reverse	5′-TTGCCAAAGACCACGTGCTT-3′		
PPIA-Probe	5′-AGACTGAGTGGTTGGATG-3′		

Regarding the gDNA yields fluorometric quantification ranged between 7.6 ng/µl and 300 ng/µl despite the DNAse incubation performed during extraction step: the unexpected high yields of residual gDNA on several tested samples were further checked by no-RT control reactions (only qPCR without RT step as previously stated), confirming presence of late Cq amplification plots from PPIA pseudogene copies that are present in bovine gDNA. However, a difference higher than 5 Cq between qPCR-only amplified RNAs and respective samples submitted to RT-qPCR was found. According to Laurell et al. (2012) the contaminant gDNA late amplification signals (where ΔCq higher than 5 Cq) do not affect the real contribution of targeted cDNA to amplification plots.

**Figure 1:**
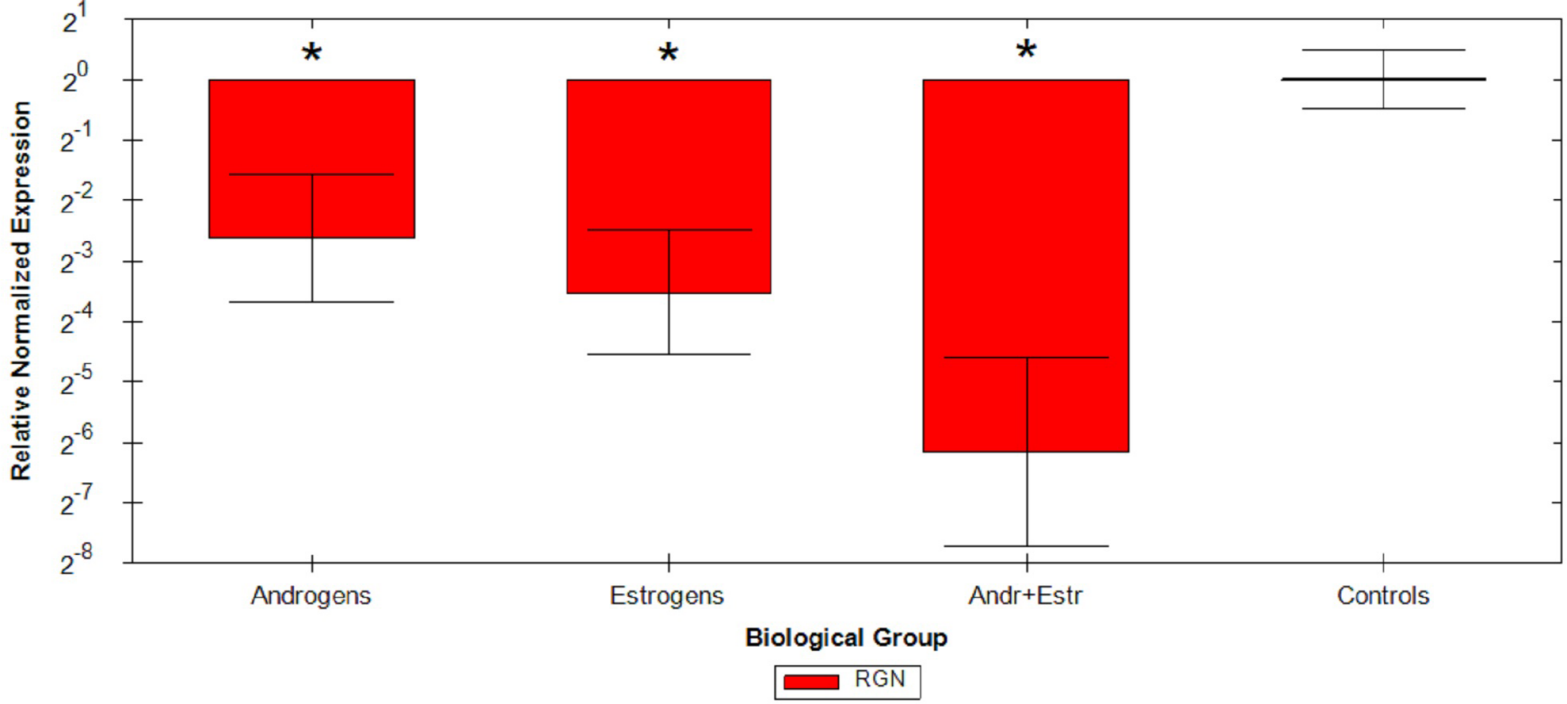
Graphical representation of steroids treatment. Graphical representation of steroids treatment down regulation on RGN relative expression by ΔΔCq method on different veal calves groups. *p < 0.05.

Relative Quantification analysis by the ΔΔCq method were then performed on all samples: comparative study confirmed that androgens cause a mean 2.62 fold reduction, estrogens cause a mean 3.52 fold reduction and the association of the two hormones causes a mean 6.16 fold reduction of RGN mRNA levels compared to untreated animals (Fig. 1 and Table 2). ANOVA results are reported in Table 3, resulting significant for all considered group comparisons.

Parameters of daily standard curves loaded in each plate together with cDNA samples are reported in Table 4, quantification of PPIA and RGN copy-number were then successfully performed referring collected Cq data to full range standard curve model: no sample analyzed was below the LOQ value (50 PPIA and 50 RGN target copies with CV% ≤ 25%). A detailed description of the full range curves and validation parameters is reported in Supplemental Information 2.

For PPIA/RGN copy-ratio calculation and ROC curve application, an optimal cut-off at 0.03118 was selected (Supplementary Material S2): at the established cut-off limit, the analysis allowed the discrimination of positive samples from negative ones with a sensitivity of 96.67% (82.78% to 99.92%, CI 95%) with a mean specificity of 90%, (55.50% to 99.75%, 95% CI, 9.667 likelihood ratio).

**Figure 2:**
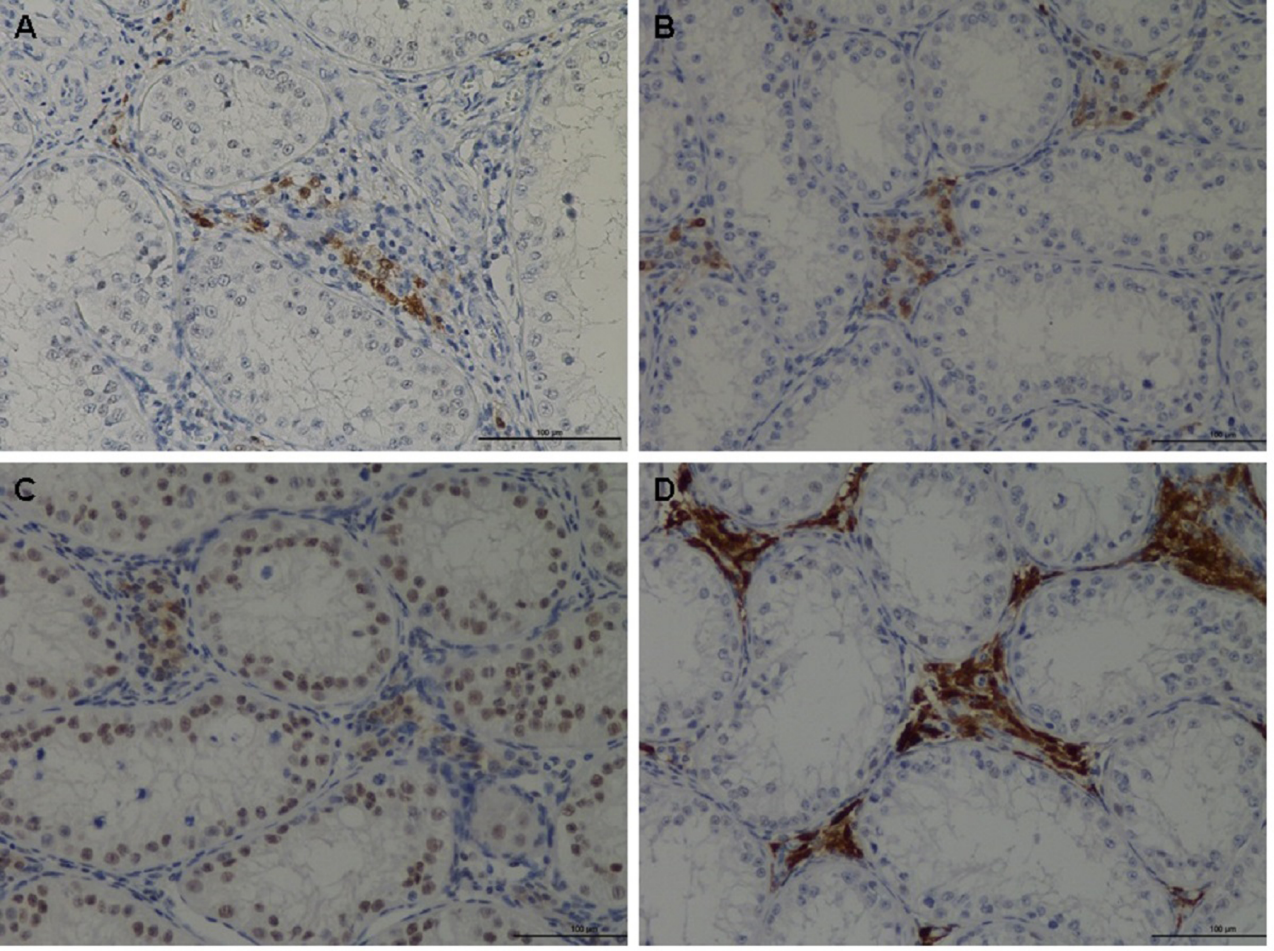
Immunohistochemical of RGN protein in the testis of veal calves. Different expression of RGN in animal treated with nandrolone (A), 17β-estradiol (B), association of nandrolone and 17β-estradiol (C) and control group (D). RGN protein was in the cytoplasm of Leydig cells although the nuclei of some spermatogonia showed staining. Bar = 100 µm.

The IHC showed that RGN protein was predominantly expressed in Leydig cells and also in a variety of germ cells (Fig. 2). Although cell localization was essentially cytoplasmic, some nuclear staining was visible particularly in the nuclei of some spermatogonia. A significant reduction (*p* < 0.0001) in RGN expression was observed in all treated animals (nandrolone, 17β estradiol and their association) compared to the control group. The results are reported in Table 5.

ROC curve was then calculated also for IHC test (Supplemental Information 4); with a cut-off of 0.0542 the performances of the test in term of sensitivity and specificity were respectively of 83.33% (range from 65.28% to 94.36%, 95% CI) and 90% (from 55.50% to 99.75%, 95% CI, 8.333 likelihood ratio).

**Figure 3:**
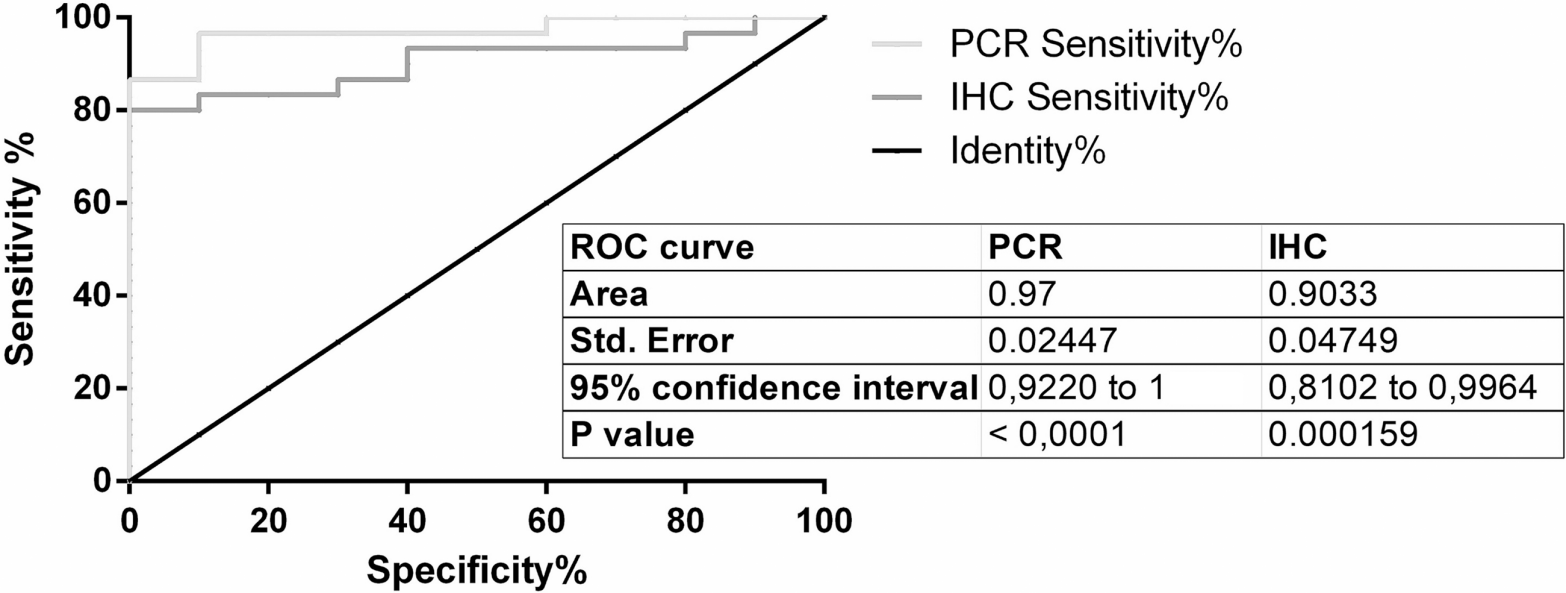
ROC curves. ROC curves of RT-qPCR and IHC test.

Graphical representation of both ROC curves was reported in Fig. 3.

**Table 2:** RGN fold regulation by different steroids tested.

Biological Group	Expression	95% CI Low	95% CI High	*p*-value
Andr+Estr	0.01393	0.00473	0.04106	0.000001
Androgens	0.16213	0.07782	0.33778	0.000050
Estrogens	0.08658	0.04225	0.17742	0.000002
Controls	1	0.71971	1.38944	N/A

**Table 3:** ANOVA comparisons of relative expression levels among treated and control veal calves groups.

*p*-value ANOVA	Comparisons	Mean Diff.	95% CI of Diff. (low to high)	*p*- value Dunnet	Significant?
	Andr+Estr-Controls	−5,465	−7,154 to −3,776	<0,01	Yes
	Androgens-Controls	−2,581	−4,317 to −0,846	<0,01	Yes
	Estrogens-Controls	−3,549	−5,284 to −1,813	<0,01	Yes
4,00^E−08^					Yes

**Table 4:** Standard curves parameters from serial ten-fold dilutions of reference plasmid material.

Target:	PPIA	RGN
Slope:	−3.40093	−3.35604
Intercept:	39.07534	39.03665
Efficiency:	0.96806	0.98596
Residual variance:	0.03163	0.01508
SE(intercept):	0.07423	0.05126
SE(slope):	0.01875	0.01294
SE(Efficiency):	0.00734	0.00526
Confidence:	95%	95%
Critical *t*-value:	2.01669	2.01669
R^2^:	0.9987	0.99936

**Table 5:** Detail of area of tissue RGN positive (expressed as a percentage) in all treated animals (nandrolone, 17β estradiol and their association) compared to the control group. Different superscript (a, b, c) indicate a statistical difference.

**Biological Group**	**Median (MIN–MAX)**	**SD**
Andr+Estr	3,17%^a^^*^(2,13%–8,66%)	2,6
Androgens	3,17%^b^^*^ (0,52%–12,04%)	4,08
Estrogens	2,24%^c^^*^ (0,48%–4,38%)	1,35
Controls	8,27% (5,22%–29,86%)	7,28

**Notes.**

* *p* ≤ 0.0001.

## Discussion

In recent years FFPE materials, collected and stored for conventional histological and IHC analysis, are becoming more and more interesting for nucleic acid analysis (Wimmer et al., 2018). Long term storage FFPE blocks have been analyzed with different molecular high-throughput techniques like microarrays and NGS revealing that, despite the known alterations in nucleic acids, suitable biological information can still be gained (SolbergEikrem et al., 2015). The possibility to exploit FFPE tissues for a diagnostic use of mRNA biomarkers in official control activities has been recently evaluated (Benedetto et al., 2020).

To further extend the applicability of FFPE samples profiling in current veterinary inspection and sampling activities, in order to improve detection of illicit treatments by growth promoters in meat production, a combined use of quantitative methods based on Immunohistochemistry and Polymerase Chain Reaction techniques was applied to RGN, an interesting biomarker responsive to sex steroids exposure recently described by Starvaggi Cucuzza et al. (2017).

RGN was first identified in 1978 as a calcium (Ca2+) binding protein (Yamaguchi & Yamamoto, 1978). RGN expression in all testicular cell types from rat, human and bovine was confirmed by PCR, Western blot and IHC (Laurentino et al., 2011; Starvaggi Cucuzza et al., 2014). In addition, steroid and non-steroid hormones have been described to regulate RGN expression, particularly the decrease of RGN gene and protein expression in bovine testis (Starvaggi Cucuzza et al., 2014).

The objective quantification of histological results is becoming essential to define strict and well established protocols. Recent studies showed indeed that quantitative data could allow a more precise correlation to clinical or biological data (Casiraghi et al., 2018).

The evaluation of IHC labelling for RGN in our study was therefore performed by automatic analysis to produce data that are more rigorous and on a continuous scale compared to qualitative and/or semiquantitative IHC approaches.

IHC quantification data and cut-off setting by ROC curve showed satisfactory performances in terms of test specificity and sensitivity, allowing an automated and cheaper analysis when compared to RT-qPCR analysis performed on the same biomarker.

On the other hand, the modifications here implemented to the original method for RGN mRNA profiling in veal calves based on RNA*later* testis samples (Starvaggi Cucuzza et al., 2017) allowed to successfully analyse also FFPE testis specimens. This outcome was achieved by redesigning assays to overcome some FFPE-related technical limitations, as confirmed at first by consistent RGN down regulation found by ΔΔCq method (Fig. 1), then by RGN/PPIA copy-ratio determination by standard curve quantification approach (Supplemental Information 2).

For example, the reduced sensitivity on FFPE extracts of the original assays, which is due to larger amplicons for both PPIA and RGN, was solved with a new design based on smaller amplicons and Taqman MGB probes.

Another unexpected finding was related to the presence of early PPIA Cq signals during the analysis of no RT controls, due to PPIA pseudogenes presence and the massive excess of gDNA in the testis RNA extracts. Such an issue seems connected to the spermatogenesis process that is known to cause a significantly different DNA:RNA ratio compared with other somatic cells (Cappallo-Obermann et al., 2011). The choice of a one-step RT-qPCR including a further step of gDNA depletion and the check of ΔCq between no-RT controls and corresponding RT-qPCR Cq values, according to Laurell et al. (2012), could guarantee reliable estimation of PPIA target copies and normalization of RQ data (by ΔΔCq method) and/or PPIA/RGN ratio calculations.

Relative quantification data collected from FFPE specimens were in agreement with the observations of previous studies (Starvaggi Cucuzza et al., 2014), maintaining comparable trends in sex steroids RGN down regulation. Indeed, from Δ ΔCq experiment data, we confirmed a strong inhibition of RGN expression induced by androgens and estrogens in veal calves’ testis, still detectable on ten year old FFPE testis samples. Moreover, the simultaneous administration of androgens and estrogens additively induce a more than six fold down regulation in treated animals.

With regard to standard curve quantifications, the approach that remains more suitable for a diagnostic use of RGN profiling as molecular test, a ROC curve-based cut-off, was chosen to define a good balance between sensitivity and specificity of the test. The identified cut-off on FFPE samples (0.03118) was higher when compared to the cut-off value found by Starvaggi Cucuzza and co-authors (2017) on RNA*later* specimens (0.01041). Causes for this discrepancy are mostly related to the effects of FFPE on RNA fragmentation, which partially reduced the possibility to detect all starting copies of PPIA and RGN in an unknown sample, influencing therefore the copy-ratio values, as shown in different studies regarding mRNA quantification in FFPE specimens (Bjorkman et al., 2015; Kap et al., 2015).

Regarding evaluation of suitable diagnostic protocols based on IHC and RT-qPCR, many statistical approaches are available to combine multiple diagnostic tests for screening purposes (Irwin & Irwin, 2011; Maxim, Niebo & Utell, 2014; Qin & Zhang, 2010). However, the parallel, serial and/or nested use of multiple tests could be shaped in current monitoring plans for growth promoter surveillance not only on the basis of their diagnostic performances, but also considering optimal sampling strategies, the expected prevalence of such illicit practices, the usability of these analyses in legal disputes and, obviously, analytical costs.

Based on the preliminary results from this study, RT-qPCR confirms RGN as useful and robust biomarkers able, together ICH analysis, to detect illegal administration of sex steroid hormones in veal calves. Specifically, RT-qPCR was more sensitive than IHC test for detection of steroids abuse in veal calves by RGN expression analysis, but also more expensive and time consuming than automated quantitative IHC analysis. The availability of these different diagnostic tests to the labs involved in official controls would allow a convenient choice based on their operational capabilities.

However, considering the potential field application of IHC and RT-qPCR for routine analyses in NRCPs, an efficient and balanced cost-benefit strategy to combine such tests will need to be further evaluated on field samples, with the aim to enlarge the numbers of screened animals/farms and, in the future, to limit confirmation analyses, usually based on more expensive analytical methods, only where needed.

## Conclusions

The study has shown a potential application of regucalcin (RGN) profiling at both transcriptional and protein level. Developed screening tests on formalin fixed paraffin embedded samples (FFPE) are suitable for monitoring activities at slaughterhouse planned according to the Italian histological monitoring plan to support official controls for sex steroid hormone misuse in calves. The flexibility of developed methods, successfully applied to ten years old FFPE blocks, could allow both retrospective analyses, when official investigations on growth promoters abuse are requested by authorities, and implementation/revision of current NRCPs.

##  Supplemental Information

10.7717/peerj.10894/supp-1 Supplemental Information 1 Raw data

10.7717/peerj.10894/supp-2 Supplemental Information 2 Reference material preparation and qPCR validation parameters (performed as already reported in (Benedetto et al., 2020))

10.7717/peerj.10894/supp-3 Supplemental Information 3 PCR cut-off by ROC curve analysis. Chosen cut-off value highlighted in bold

10.7717/peerj.10894/supp-4 Supplemental Information 4 IHC cut-off by ROC curve analysis. Chosen cut-off value highlighted in bold

## Additional Information and Declarations

### Data Availability

The following information was supplied regarding data availability: Raw data is available in the Supplemental Files.
