# Supplementary material for: Regucalcin expression profiles in veal calf testis: validation of histological and molecular tests to detect sex steroids illicit administration
Source: PeerJ. 2021 Feb 19;9:e10894. doi: 10.7717/peerj.10894 (PMC7899017; doi:10.7717/peerj.10894)
Supplement: Supplemental Information 2 [file peerj-09-10894-s002.docx]

**Supplementary Material 1**

**Reference material preparation and qPCR validation parameters** (performed as already reported in the paper of Benedetto et al., 2020 cited in the manuscript).

An artificial DNA template containing the sequence of RGN, PPIA and other amplicons, some of them used for validation of assays already published by our team (see validation of PPIA and PgR assay reported in Benedetto et al., 2020) was produced by multiple overlapping PCR method and cloned into pDRIVE plasmid (Qiagen) following the manufacture’s guidelines. Plasmids batches carrying the fusion amplicons were checked by Sanger sequencing and they were then linearized by SmaI digestion (Promega), quantified by Qubit dsDNA fluorimetric assay (ThermoFisher) and submitted to a first trial of standard curves experiments (four separate runs for both multiplexed targets performed in different days, five replicates for each dilutions). This reference material for full range standard curve set was prepared by five fold volumetric dilutions to cover a broad concentrations range (nine serial dilutions from 5*10^5^ to ≈1 nominal target copy) and to check some validation parameters: linear range, coefficient of correlation, efficiency, LOD and LOQ.

Finally, for the quantitative analysis of FFPE samples, daily standard curve based on five ten-fold serial dilutions (from 5*10^5^ to 50 copies of both target, three replicate for each dilutions) was loaded on each 96 well Real Time optical plates (Biorad) used for analysis on FFPE cDNA samples, in order to ensure quantification of RGN and PPIA transcript and to grant optimal interplate calibrations when referring to the full range standard curves.

Basic validation parameters were then checked as recommended by Breoders et al., (2014):

- Assays specificity (*in silico* by BLAST alignment, experimentally by analysis on NTC and other non-target DNA material)

- Assays linearity (R^2^ ≥ 0.98) and PCR efficiency (ranging from 90 to 110%)

- Limit of Detection (LOD) merging full range standard curve data points (8 five-fold serial dilutions from 5*10^5^ to ≈6 copies) to the daily standard curves loaded in each PCR plate (5 ten-fold serial dilutions used for the interplate calibration)

- Limit of Quantification (LOQ as LOD value where CV% ≤ 25%), calculated on the daily curves

- Robustness verified by stressing method parameters and run conditions, similarly to Youden’s combination test criteria adopted in analytical chemistry: ±1°C of optimal annealing temperatures, -30% of primer concentrations and ±1µL of 2x Master mix from what reported in the paper (see Material and Methods - Multiplex One step RT-qPCR)

Analysis of validation parameters where performed by GenEx software ver. 6.1 (MultiID) merging all daily standard curves and full range standard curves. Moreover the software allows the application of CLSI:EP6-A criteria for the evaluation of quantitative measurement. For LOD and LOQ evaluation the software applied the method described by Forootan et al., (2017).

**Results**

The specificity of the assays was confirmed *in silico* on *Bos taurus* transcriptome by checking selectivity for bovine RGN and PPIA mRNA, and moreover by testing non-target DNA that might be present in the reagents used for method development (Sheared Salmon Sperm DNA used for plasmid dilutions).

Assays efficiencies, curve slopes, intercept and R^2^ values, assessed by analysis on full range standard curves are reported in the following table, showing acceptable overlapping with the daily curves (see table 4 of the manuscript):

| Curves | RGN | | | | | PPIA | | | | |
| --- | --- | --- | --- | --- | --- | --- | --- | --- | --- | --- |
| Slope: | -3.37808 | < | -3.34596 | < | -3.31385 | -3.40913 | < | -3.37036 | < | -3.33159 |
| Intercept: | 39.26336 | < | 39.37978 | < | 39.49621 | 38.69177 | < | 38.83234 | < | 38.97291 |
| Efficiency: | 0.97692 | < | 0.99007 | < | 1.00321 | 0.96462 | < | 0.98018 | < | 0.99574 |
| Residual variance: | 0.13604 |  |  |  |  | 0.19831 |  |  |  |  |
| SE(intercept): | 0.05904 |  |  |  |  | 0.07128 |  |  |  |  |
| SE(slope): | 0.01628 |  |  |  |  | 0.01966 |  |  |  |  |
| SE(Efficiency): | 0.00667 |  |  |  |  | 0.00789 |  |  |  |  |
| Confidence: | 95% |  |  |  |  | 95% |  |  |  |  |
| Critical t-value: | 1.97202 |  |  |  |  | 1.97202 |  |  |  |  |
| R^2^ | 0.99533 |  |  |  |  | 0.99331 |  |  |  |  |

LOD values were 8.9 copies for RGN and 6.8 copies for PPIA assay respectively, with a 95% confidence level. Graphical representation of LOD as fractions of positive reads obtained by RGN and PPIA assays fitted to a sigmoidal curve is reported as follows.


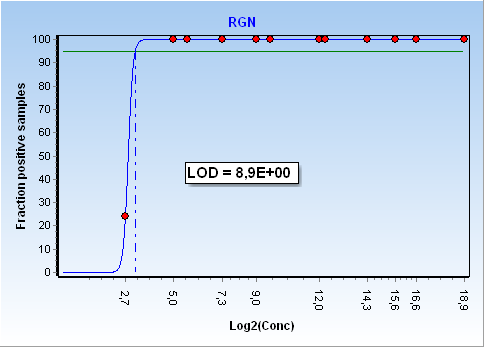

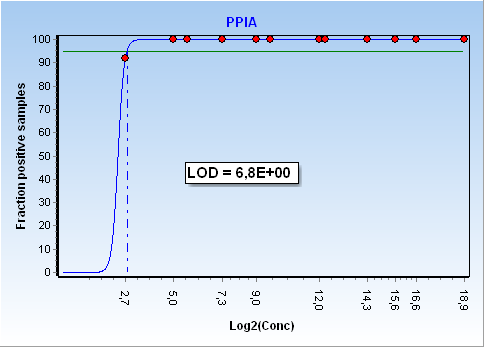


LOQ values on daily plates ten-fold dilutions standards curve was 50 copies for both RGN PPIA assay (with acceptable inter-assay CV% of 25%, see plotted CV% values below).


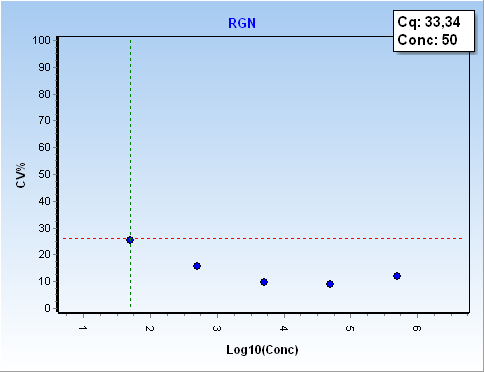

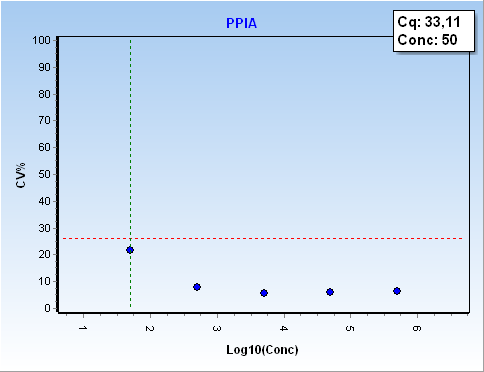


CLSI:EP6-A criteria, verified by automatic calculations performed by GenEx software, resulted satisfied for both RGN and PPIA standard curves.

| CLSI:EP6-A | RGN | | | CLSI:EP6-A | PPIA | | |
| --- | --- | --- | --- | --- | --- | --- | --- |
| Deg 1 coefficients: |  |  |  | Deg 1 coefficients: |  |  |  |
|  | 39.37978 |  |  |  | 38.83234 |  |  |
|  | -3.34596 |  |  |  | -3.37036 |  |  |
| Standard error: | 0.36883 |  |  | Standard error: | 0.44532 |  |  |
| Coeff standard error: | 0.05904 | t-test: | 667.00775 | Coeff standard error: | 0.07128 | t-test: | 544.76319 |
| Coeff standard error: | 0.01628 | t-test: | -205.46858 | Coeff standard error: | 0.01966 | t-test: | -171.41824 |
|  |  |  |  |  |  |  |  |
| Deg 2 coefficients: |  |  |  | Deg 2 coefficients: |  |  |  |
|  | 39.42977 |  |  |  | 39.02407 |  |  |
|  | -3.38654 |  |  |  | -3.52599 |  |  |
|  | 0.00624 |  |  |  | 0.02392 |  |  |
| Standard error: | 0.3695 |  |  | Standard error: | 0.44317 |  |  |
| Coeff standard error: | 0.11066 | t-test: | 356.31476 | Coeff standard error: | 0.13272 | t-test: | 294.02265 |
| Coeff standard error: | 0.07765 | t-test: | -43.61372 | Coeff standard error: | 0.09313 | t-test: | -37.86054 |
| Coeff standard error: | 0.01167 | t-test: | 0.53447 | Coeff standard error: | 0.014 | t-test: | 1.70924 |
|  |  |  |  |  |  |  |  |
| Deg 3 coefficients: |  |  |  | Deg 3 coefficients: |  |  |  |
|  | 39.28754 |  |  |  | 39.0961 |  |  |
|  | -3.19014 |  |  |  | -3.62545 |  |  |
|  | -0.06417 |  |  |  | 0.05958 |  |  |
|  | 0.00722 |  |  |  | -0.00365 |  |  |
| Standard error: | 0.36982 |  |  | Standard error: | 0.44417 |  |  |
| Coeff standard error: | 0.20725 | t-test: | 189.56976 | Coeff standard error: | 0.24891 | t-test: | 157.0677 |
| Coeff standard error: | 0.25407 | t-test: | -12.55628 | Coeff standard error: | 0.30515 | t-test: | -11.88099 |
| Coeff standard error: | 0.0875 | t-test: | -0.7334 | Coeff standard error: | 0.10509 | t-test: | 0.56693 |
| Coeff standard error: | 0.00889 | t-test: | 0.81195 | Coeff standard error: | 0.01067 | t-test: | -0.34234 |
|  |  |  |  |  |  |  |  |
| RandomError: | 0.37266 |  |  | RandomError: | 0.44833 |  |  |
| RandomErrorRel: | 1.058 |  |  | RandomErrorRel: | 1.28218 |  |  |
| Result: | Data is linear | | | Result: | Data is linear | | |

Robustness, tested as previously reported with small deviations in primer concentrations, mastermix volumes and annealing temperature variations was fully confirmed (Cq deviations induced < Cq SD).

Quantification on FFPE samples were performed by conventional reverse calibration against broad range standard curve model, using for interplate calibration the daily standard curves loaded in each PCR plate as previously reported. The overlap of all replicates of both five dilution points daily curves (red dots) and full range quantification curves (green dots) are here represented together with residual plots of replicates distribution (no outliers removed).


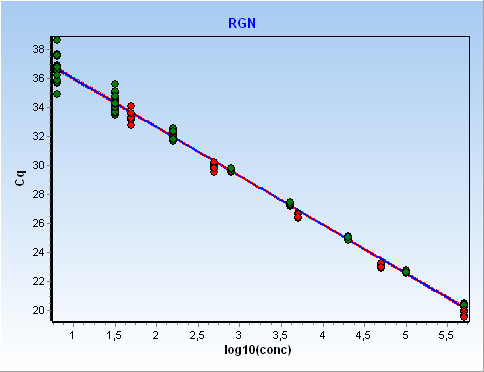

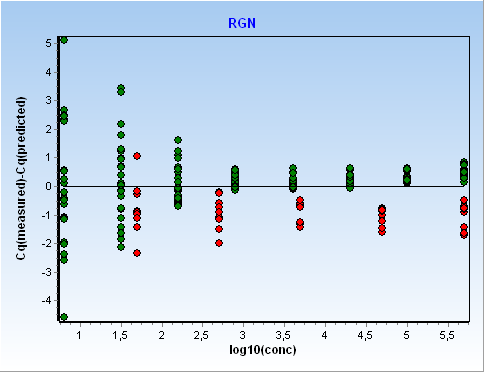


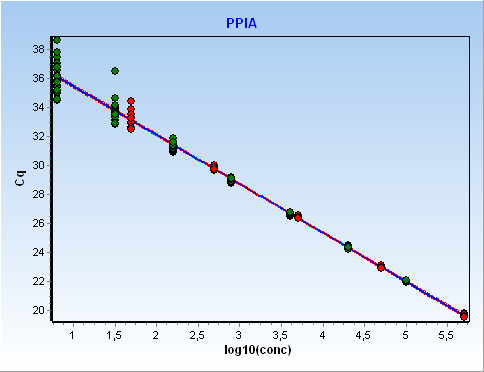

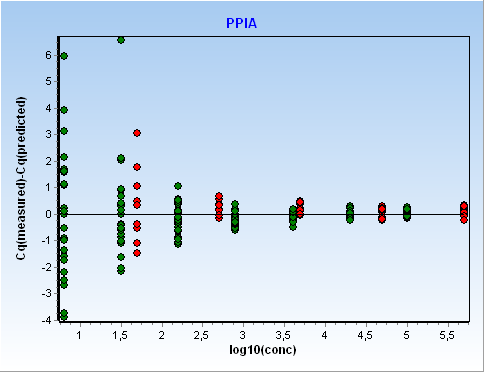


**Bibliography**

Benedetto A., Pezzolato M., Beltramo C., Audino V., Ingravalle F., Pillitteri C., Foschini S., Peletto S., Bozzetta E. (2020). Real-time PCR assay for detecting illicit steroid administration in veal calves allows reliable biomarker profiling of formalin-fixed, paraffin-embedded (FFPE) archival tissue samples. Food Chem 312:126061.

Broeders, S., Huber, I., Grohmann, L., Berben, G., Taverniers, I., Mazzara, M., Roosens, N., & Morisset, D. (2014). Guidelines for validation of qualitative real-time PCR methods. Trends in Food Science & Technology, 37(2), 115-126.

Forootan, A., Sjoback, R., Bjorkman, J., Sjogreen, B., Linz, L., & Kubista, M. (2017). Methods to determine limit of detection and limit of quantification in quantitative real-time PCR (qPCR). Biomol Detect Quantif, 12, 1-6.
