# Supplementary material for: Regucalcin expression profiles in veal calf testis: validation of histological and molecular tests to detect sex steroids illicit administration
Source: PeerJ. 2021 Feb 19;9:e10894. doi: 10.7717/peerj.10894 (PMC7899017; doi:10.7717/peerj.10894)
Supplement: Supplemental Information 3 [file peerj-09-10894-s003.docx]

Supplementary Material 2: PCR cut-off by ROC curve analysis. Chosen cut-off value highlighted in **bold**

| Cut-off | Sensitivity% | 95% CI | Specificity% | 95% CI | Likelihood ratio |
| --- | --- | --- | --- | --- | --- |
| < 0.001038 | 3.333 | 0.08436% to 17.22% | 100 | 69.15% to 100% |  |
| < 0.002234 | 6.667 | 0.8178% to 22.07% | 100 | 69.15% to 100% |  |
| < 0.003468 | 10 | 2.112% to 26.53% | 100 | 69.15% to 100% |  |
| < 0.004117 | 13.33 | 3.755% to 30.72% | 100 | 69.15% to 100% |  |
| < 0.004761 | 16.67 | 5.642% to 34.72% | 100 | 69.15% to 100% |  |
| < 0.005446 | 20 | 7.713% to 38.57% | 100 | 69.15% to 100% |  |
| < 0.006243 | 23.33 | 9.934% to 42.28% | 100 | 69.15% to 100% |  |
| < 0.006848 | 26.67 | 12.28% to 45.89% | 100 | 69.15% to 100% |  |
| < 0.007360 | 30 | 14.73% to 49.40% | 100 | 69.15% to 100% |  |
| < 0.007759 | 33.33 | 17.29% to 52.81% | 100 | 69.15% to 100% |  |
| < 0.008704 | 36.67 | 19.93% to 56.14% | 100 | 69.15% to 100% |  |
| < 0.009623 | 40 | 22.66% to 59.40% | 100 | 69.15% to 100% |  |
| < 0.01058 | 43.33 | 25.46% to 62.57% | 100 | 69.15% to 100% |  |
| < 0.01166 | 46.67 | 28.34% to 65.67% | 100 | 69.15% to 100% |  |
| < 0.01187 | 50 | 31.30% to 68.70% | 100 | 69.15% to 100% |  |
| < 0.01275 | 53.33 | 34.33% to 71.66% | 100 | 69.15% to 100% |  |
| < 0.01366 | 56.67 | 37.43% to 74.54% | 100 | 69.15% to 100% |  |
| < 0.01381 | 60 | 40.60% to 77.34% | 100 | 69.15% to 100% |  |
| < 0.01425 | 63.33 | 43.86% to 80.07% | 100 | 69.15% to 100% |  |
| < 0.01478 | 66.67 | 47.19% to 82.71% | 100 | 69.15% to 100.0% |  |
| < 0.01503 | 70 | 50.60% to 85.27% | 100 | 69.15% to 100.0% |  |
| < 0.01541 | 73.33 | 54.11% to 87.72% | 100 | 69.15% to 100.0% |  |
| < 0.01604 | 76.67 | 57.72% to 90.07% | 100 | 69.15% to 100.0% |  |
| < 0.01736 | 80 | 61.43% to 92.29% | 100 | 69.15% to 100.0% |  |
| < 0.01906 | 83.33 | 65.28% to 94.36% | 100 | 69.15% to 100.0% |  |
| < 0.02034 | 86.67 | 69.28% to 96.24% | 100 | 69.15% to 100.0% |  |
| < 0.02325 | 86.67 | 69.28% to 96.24% | 90 | 55.50% to 99.75% | 8.667 |
| < 0.02568 | 90 | 73.47% to 97.89% | 90 | 55.50% to 99.75% | 9 |
| < 0.02759 | 93.33 | 77.93% to 99.18% | 90 | 55.50% to 99.75% | 9.333 |
| **< 0.03118** | **96.67** | **82.78% to 99.92%** | **90** | **55.50% to 99.75%** | **9.667** |
| < 0.03478 | 96.67 | 82.78% to 99.92% | 80 | 44.39% to 97.48% | 4.833 |
| < 0.03731 | 96.67 | 82.78% to 99.92% | 70 | 34.75% to 93.33% | 3.222 |
| < 0.04242 | 96.67 | 82.78% to 99.92% | 60 | 26.24% to 87.84% | 2.417 |
| < 0.04846 | 96.67 | 82.78% to 99.92% | 50 | 18.71% to 81.29% | 1.933 |
| < 0.05400 | 96.67 | 82.78% to 99.92% | 40 | 12.16% to 73.76% | 1.611 |
| < 0.05964 | 100 | 88.43% to 100.0% | 40 | 12.16% to 73.76% | 1.667 |
| < 0.06760 | 100 | 88.43% to 100.0% | 30 | 6.674% to 65.25% | 1.429 |
| < 0.07839 | 100 | 88.43% to 100.0% | 20 | 2.521% to 55.61% | 1.25 |
| < 0.09245 | 100 | 88.43% to 100.0% | 10 | 0.2529% to 44.50% | 1.111 |
