# Supplementary material for: Regucalcin expression profiles in veal calf testis: validation of histological and molecular tests to detect sex steroids illicit administration
Source: PeerJ. 2021 Feb 19;9:e10894. doi: 10.7717/peerj.10894 (PMC7899017; doi:10.7717/peerj.10894)
Supplement: Supplemental Information 4 [file peerj-09-10894-s004.docx]

Supplementary Material 3: IHC cut-off by ROC curve analysis. Chosen cut-off value highlighted in **bold**

| Cutoff | Sensitivity% | 95% CI | Specificity% | 95% CI | Likelihood ratio |
| --- | --- | --- | --- | --- | --- |
| < 0.00545 | 3.333 | 0.08436% to 17.22% | 100.0 | 69.15% to 100.0% |  |
| < 0.00755 | 6.667 | 0.8178% to 22.07% | 100.0 | 69.15% to 100.0% |  |
| < 0.0092 | 10.00 | 2.112% to 26.53% | 100.0 | 69.15% to 100.0% |  |
| < 0.00945 | 13.33 | 3.755% to 30.72% | 100.0 | 69.15% to 100.0% |  |
| < 0.00985 | 16.67 | 5.642% to 34.72% | 100.0 | 69.15% to 100.0% |  |
| < 0.0110 | 20.00 | 7.713% to 38.57% | 100.0 | 69.15% to 100.0% |  |
| < 0.01375 | 23.33 | 9.934% to 42.28% | 100.0 | 69.15% to 100.0% |  |
| < 0.0158 | 26.67 | 12.28% to 45.89% | 100.0 | 69.15% to 100.0% |  |
| < 0.0186 | 30.00 | 14.73% to 49.40% | 100.0 | 69.15% to 100.0% |  |
| < 0.0213 | 33.33 | 17.29% to 52.81% | 100.0 | 69.15% to 100.0% |  |
| < 0.0219 | 36.67 | 19.93% to 56.14% | 100.0 | 69.15% to 100.0% |  |
| < 0.02345 | 40.00 | 22.66% to 59.40% | 100.0 | 69.15% to 100.0% |  |
| < 0.02465 | 43.33 | 25.46% to 62.57% | 100.0 | 69.15% to 100.0% |  |
| < 0.0256 | 46.67 | 28.34% to 65.67% | 100.0 | 69.15% to 100.0% |  |
| < 0.0266 | 50.00 | 31.30% to 68.70% | 100.0 | 69.15% to 100.0% |  |
| < 0.02715 | 53.33 | 34.33% to 71.66% | 100.0 | 69.15% to 100.0% |  |
| < 0.0302 | 56.67 | 37.43% to 74.54% | 100.0 | 69.15% to 100.0% |  |
| < 0.03325 | 60.00 | 40.60% to 77.34% | 100.0 | 69.15% to 100.0% |  |
| < 0.03365 | 63.33 | 43.86% to 80.07% | 100.0 | 69.15% to 100.0% |  |
| < 0.03685 | 66.67 | 47.19% to 82.71% | 100.0 | 69.15% to 100.0% |  |
| < 0.0403 | 70.00 | 50.60% to 85.27% | 100.0 | 69.15% to 100.0% |  |
| < 0.0409 | 73.33 | 54.11% to 87.72% | 100.0 | 69.15% to 100.0% |  |
| < 0.0441 | 76.67 | 57.72% to 90.07% | 100.0 | 69.15% to 100.0% |  |
| < 0.04892 | 80.00 | 61.43% to 92.29% | 100.0 | 69.15% to 100.0% |  |
| < 0.05102 | 80.00 | 61.43% to 92.29% | 90.00 | 55.50% to 99.75% | 8.000 |
| **< 0.0542** | **83.33** | **65.28% to 94.36%** | **90.00** | **55.50% to 99.75%** | **8.333** |
| < 0.06009 | 83.33 | 65.28% to 94.36% | 80.00 | 44.39% to 97.48% | 4.167 |
| < 0.06394 | 83.33 | 65.28% to 94.36% | 70.00 | 34.75% to 93.33% | 2.778 |
| < 0.06581 | 86.67 | 69.28% to 96.24% | 70.00 | 34.75% to 93.33% | 2.889 |
| < 0.06906 | 86.67 | 69.28% to 96.24% | 60.00 | 26.24 to 87.84% | 2.167 |
| < 0.0729 | 90.00 | 73.47% to 97.89% | 60.00 | 26.24% to 87.84% | 2.250 |
| < 0.07497 | 93.33 | 77.93% to 99.18% | 60.00 | 26.24% to 87.84% | 2.333 |
| < 0.0788 | 93.33 | 77.93% to 99.18% | 50.00 | 18.71% to 81.29% | 1.867 |
| < 0.08379 | 93.33 | 77.93% to 99.18% | 40.00 | 12.16% to 73.76% | 1.556 |
| < 0.09140 | 93.33 | 77.93% to 99.18% | 30.00 | 6.674% to 65.25% | 1.333 |
| < 0.09834 | 93.33 | 77.93% to 99.18% | 20.00 | 2.521% to 55.61% | 1.167 |
| < 0.1052 | 96.67 | 82.78% to 99.92% | 20.00 | 2.521% to 55.61% | 1.208 |
| < 0.1140 | 96.67 | 82.78% to 99.92% | 10.00 | 0.2529% to 44.50% | 1.074 |
| < 0.2114 | 100.0 | 88.43% to 100.0% | 10.00 | 0.2529% to 44.50% | 1.111 |
